# Retraction: Sensitive Detection of Pre-Existing BCR-ABL Kinase Domain Mutations in CD34+ Cells of Newly Diagnosed Chronic-Phase Chronic Myeloid Leukemia Patients Is Associated with Imatinib Resistance: Implications in the Post-Imatinib Era

**DOI:** 10.1371/journal.pone.0301946

**Published:** 2024-04-03

**Authors:** The PLOS ONE Editors

Following the publication of this article, concerns were raised regarding Figure 1. Specifically:

- In Figure 1A:
   ⚬ Lanes 1–3 appear similar to lanes 4–6.
   ⚬ The lanes within this panel appear similar to Fig 1 of [2] where lane conditions are described differently to the respective lanes in [1].
- In Figure 1B:
   ⚬ Lane 1 and lane 2 appear similar
   ⚬ Lanes 7–10 appear similar.

The lanes within this panel appear similar to Figure 1B of [3] where lane conditions are described differently to the respective lanes in [1].

- In Figure 1C:
   ⚬ Each lane in lanes 1–5 and 7–11 appear similar to one another.
   ⚬ There appear to be vertical discontinuities between lanes 5 and 6 and lanes 6 and 7.

The corresponding author stated the original underlying data were no longer available for editorial review. In the absence of original underlying data, these concerns cannot be resolved.

In light of the concerns affecting multiple panels in Figure 1 that question the integrity and reliability of these data, the *PLOS ONE* Editors retract this article.

ZI and AA did not agree with the retraction. MI, MIN, AG, AST, AQ, NuR, AMK, HIS, MK, RH, MK, SMB, AJ, MNA, MA, AM, MR, and TA either did not respond directly or could not be reached.
